# Immuno-Stimulatory Activity of *Escherichia coli* Mutants Producing Kdo_2_-Monophosphoryl-Lipid A or Kdo_2_-Pentaacyl-Monophosphoryl-Lipid A

**DOI:** 10.1371/journal.pone.0144714

**Published:** 2015-12-28

**Authors:** Biwen Wang, Yaning Han, Ye Li, Yanyan Li, Xiaoyuan Wang

**Affiliations:** 1 State Key Laboratory of Food Science and Technology, Jiangnan University, Wuxi, 214122, China; 2 School of Food Science and Technology, Jiangnan University, Wuxi, 214122, China; 3 Synergetic Innovation Center of Food Safety and Nutrition, Jiangnan University, Wuxi, China; Instituto Butantan, BRAZIL

## Abstract

Lipid A is the active center of lipopolysaccharide which also known as endotoxin. Monophosphoryl-lipid A (MPLA) has less toxicity but retains potent immunoadjuvant activity; therefore, it can be developed as adjuvant for improving the strength and duration of the immune response to antigens. However, MPLA cannot be chemically synthesized and can only be obtained by hydrolyzing lipopolysaccharide (LPS) purified from Gram-negative bacteria. Purifying LPS is difficult and time-consuming and can damage the structure of MPLA. In this study, *Escherichia coli* mutant strains HWB01 and HWB02 were constructed by deleting several genes and integrating *Francisella novicida* gene *lpxE* into the chromosome of *E*. *coli* wild type strain W3110. Compared with W3110, HWB01 and HWB02 synthesized very short LPS, Kdo_2_-monophosphoryl-lipid A (Kdo_2_-MPLA) and Kdo_2_-pentaacyl-monophosphoryl-lipid A (Kdo_2_-pentaacyl-MPLA), respectively. Structural changes of LPS in the outer membranes of HWB01 and HWB02 increased their membrane permeability, surface hydrophobicity, auto-aggregation ability and sensitivity to some antibiotics, but the abilities of these strains to activate the TLR4/MD-2 receptor of HKE-Blue hTLR4 cells were deceased. Importantly, purified Kdo_2_-MPLA and Kdo_2_-pentaacyl-MPLA differed from wild type LPS in their ability to stimulate the mammalian cell lines THP-1 and RAW264.7. The purification of Kdo_2_-MPLA and Kdo_2_-pentaacyl-MPLA from HWB01 and HWB02, respectively, is much easier than the purification of LPS from W3110, and these lipid A derivatives could be important tools for developing future vaccine adjuvants.

## Introduction

**Fig 1:**
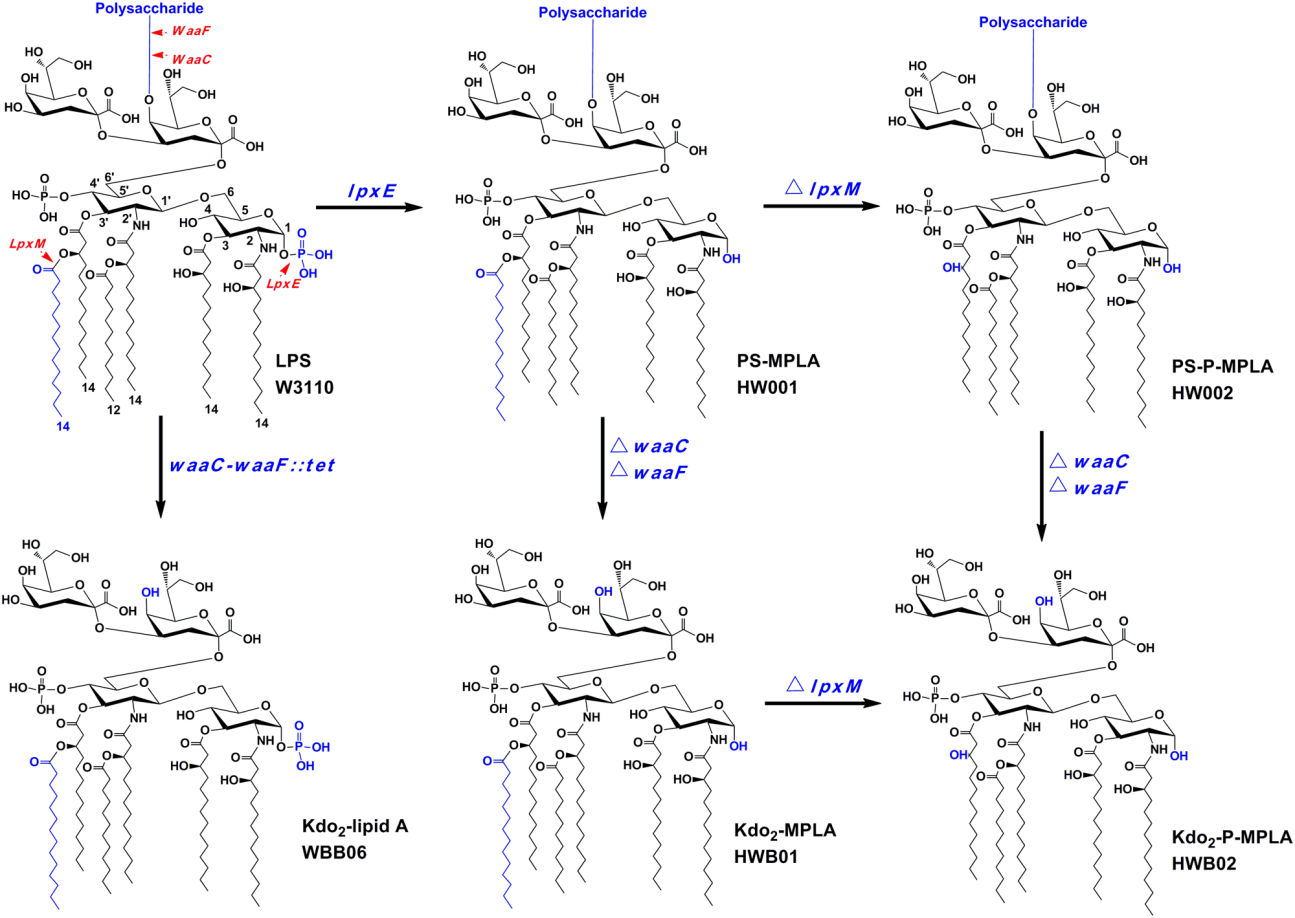
LPS structure comparison of *E*. *coli* strains W3110, HW001, HW002, WBB06, HWB01 and HWB02. Red numbers specify the glucosamine ring positions, and the black numbers indicate the predominant fatty acid chain lengths. The enzymes WaaC, WaaF, LpxM and LpxE are shown in res with arrows indicating their functional domains, and the groups removed by these enzymes are shown in blue. Expression of *lpxE* in W3110 forms the strain HW001 that synthesizes PS-MPLA, and deletion of *lpxM* in HW001 forms the strain HW002 that synthesizes PS-pentaacyl-MPLA. Inactivation of *waaC* and *waaF* in W3110, HW001 and HW002 resulted in strains WBB06, HWB01 and HWB02, respectively. WBB06, HWB01, and HWB02 synthesize Kdo_2_-lipid A, Kdo_2_-MPLA and Kdo_2_-pentaacyl-MPLA, respectively.

Lipopolysaccharide (LPS), the major component of the outer layer of outer membrane in most Gram-negative bacteria, is also known as endotoxin [1,2] and is important for membrane stability [3]. LPS of wild type *Escherichia coli* usually consists of Kdo_2_-lipid A and a polysaccharide (Fig 1). The lipid A moiety of LPS can be recognized by the Toll-like receptor 4 (TLR4)/myeloid differentiation protein 2 (MD-2) complex that activates the mammalian innate immune system and results in the production of potent inflammation mediators, such as interleukin-6 (IL-6), tumor necrosis factor-α (TNF-α), and interleukin-8 (IL-8) [4–6]. The inflammatory toxicity of LPS is driven by either Toll-interleukin 1 receptor domain-containing adapter inducing interferon-β (TRIF) or the adaptor protein myeloid differentiation factor 88 (MyD88). Native lipid A is hexaacylated and bisphosphorylated, it can efficiently activate TLR4/MD-2 to induce leukocyte activation, cytokine production, and inflammation. This is beneficial for host because local bacterial infections can be cleared, but if these mediators are overproduced, they can damage small blood vessels and cause shock. Interestingly, after prior exposure to LPS the host becomes more resistant to subsequent LPS challenge [7]; therefore, lipid A could serve as a potent vaccine adjuvant. Native lipid A cannot be used as an immunomodulator because it can cause pronounced inflammatory responses, but certain lipid A analogs such as monophosphoryl lipid A retain some immunomodulatory properties while possessing attenuated proinflammatory activity [8,9].

The acyloxyacyl chains and phosphate groups of lipid A are important for triggering full TLR4/MD-2 activation. Native lipid A contains two phosphate groups: one is glycosidically attached to 1-position of glucosamine, and the other is an ester-bond phosphate group attached to 4’-position of distal beta-glucosamine. The loss of the phosphate group at 1-position of lipid A, resulting in monophosphoryl-lipid A (MPLA), produces a molecule with decreased affinity to the TLR4/MD-2 complex and the ability to selectively activate the TRIF signaling pathway, reducing the secretion of cytokines [10–12]. Compared to native lipid A, MPLA exhibits approximately 1/1,000th of the toxicity, but retains activity of immunoadjuvant; therefore, it has potential to be developed as vaccine adjuvant. MPLA, the low-toxicity derivative of LPS, cannot be chemically synthesized. It can only be obtained by hydrolyzing LPS, but this method is time-consuming and might damage its structure [13–15].

The biosynthesis of LPS has been well-characterized in *E*. *coli*, and several lipid A modification enzymes have been reported [16]. Therefore, the structure of *E*. *coli* lipid A can now be altered by heterologous expression of these modification enzymes [17]. We have constructed *E*. *coli* mutant strains HW001 and HW002 that can synthesize LPS containing MPLA [18,19]. In the chromosome of HW001, *lacI* was deleted, and *lacZ* was replaced with *Francisella novicida lpxE*. Because LpxE effectively removes the 1-phosphate of lipid A [20], HW001 only synthesizes LPS with MPLA (PS-MPLA) (Fig 1). Deletion of *lpxM* in the chromosome of HW001 resulted in HW002. Because LpxM adds the secondary acyl chain to lipid A at 3’-position [21], HW002 synthesizes only pentaacylated MPLA (PS-pentaacyl-MPLA) (Fig 1). When stimulating murine macrophage RAW264.7 cells, PS-MPLA purified from HW001 resulted in the release of less TNF-α than W3110 LPS, and PS-pentaacyl-MPLA purified from HW002 induced less TNF-α than PS-MPLA (15).

It is not possible to construct *E*. *coli* that directly synthesizes MPLA because these cells cannot survive. Kdo_2_-lipid A is the minimal structure of LPS to sustain the bacterial viability [22–24]. The Kdo_2_-lipid A-producing *E*. *coli* strain WBB06 was constructed by inactivating genes *waaC* and *waaF* (Fig 1). The proteins encoded by these genes, WaaC and WaaF, sequentially add two L-D-heptoses to the inner Kdo residue of Kdo_2_-lipid A [21,25].

In this work, the *E*. *coli* mutant strains HWB01 and HWB02 were constructed by deleting genes *waaC* and *waaF* in the chromosomes of HW001 and HW002, respectively. Kdo_2_-MPLA and Kdo_2_-pentaacyl- MPLA were synthesized by HWB01 and HWB02, respectively (Fig 1). These molecules could be directly purified from cells without hydrolyzing. Kdo_2_-MPLA and Kdo_2_-pentaacyl-MPLA were less stimulatory towards TLR4/MD-2 than wild type LPS, suggesting these molecules could be potential novel vaccine adjuvants.

## Materials and Methods

### Construction of *E*. *coli* mutant strains

Table 1 lists all of the strains and plasmids used in this work. *E*. *coli* strains without plasmids were grown in LB medium (10 g/L tryptone, 5 g/L yeast extract and 10 g/L NaCl) at 37°C, and *E*. *coli* strains harboring plasmids pCP20 or pKD46 were grown at 30°C. When necessary, 30 μg/mL kanamycin or 100 μg/mL ampicillin was included in the medium.

**Table 1:** Bacterial strains and plasmids used in this study.

Strains or Plasmids	Description	Source
**Strains**		
W3110	Wild type *E*. *coli*	ATCC
WBB06	W3110 *waaC*-*waaF*::*tet6*	[26]
HW001	W3110 Δ*lacI lacZ*::*lpxE*	[15]
HW002	W3110 Δ*lacI* Δ*lpxM lacZ*::*lpxE*	[15]
HWB01	W3110 Δ*lacI* Δ*waaC-waaF lacZ*::*lpxE*	This work
HWB02	W3110 Δ*lacI* Δ*lpxM* Δ*waaC-waaF lacZ*::*lpxE*	This work
**Plasmids**		
pKD46	P_araB_γ β exo, Rep^ts^,Amp^R^	[52]
pKD13	FRT-kan-FRT, Amp^R^, Kan^R^	[52]
pCP20	FLP^+^, λ cI857^+^, λp_R_Rep^ts^, Cam^R^, Amp^R^	[52]
pBlueScript II SK+	Cloning vector, ColE1, *lacZ*, Amp^R^	Stratagene
pBS-CFkan	Plasmid for deleting *waaC* and *waaF* in *E*. *coli*	This work

*E*. *coli* W3110 is a nonpathogenic wild type K-12 strain and has been widely used in laboratory for gene engineering. Strain HW001 was constructed from W3110 by integrating *F*. *novicida lpxE* into *lacZ* in the chromosome, and HW002 was constructed from HW001 by deleting *lpxM* from the chromosome [19]. Because *lpxE* was integrated in the *lacZ* site, *lacI* was also removed in HW001 and HW002 to release the control to the lactose operon. *E*. *coli* strain WBB06 was derived from W3110 by inserting *tet6* into the region of *waaC* and *waaF*; this strain can only synthesize Kdo_2_-lipid A [26]. HWB01 was constructed from HW001 by deleting *waaC* and *waaF* from the chromosome, and HWB02 was constructed from HW002 by deleting *waaC* and *waaF* from the chromosome (Fig 1). Briefly, the upstream fragment of *waaC* was PCR-amplified using primers *WaaC*-U-F (5’-CCGCTCGAGTAAATCAAGCAAGCCTAT-3’) and *WaaC*-U-R (5’-AAAACTGCAGCTGCTTGCCCTGTATGGT-3’), and the downstream fragment of *waaF* was PCR amplified using primers *WaaF*-D-F (5’-CCCAAGCTTAGCTCTTATGCGTCGCGATTCAG-3’) and *WaaF*-D-R (5’-AAAACTGCAGTGCTACGCTGGCTTATC-3’). A DNA fragment containing the kanamycin resistance gene, FRT-*kan*-FRT, was amplified from pKD13 using primers *kan*-FRT-F (5’-AACTGCAGGTGTAGGCTGGAGCTGCTTCG-3’) and *kan*-FRT-R (5’-CCCAAGCTTACCTGCAGTTCGAAGTTCCT-3’). These three fragments were then ligated together to form plasmid pBS-CFkan carrying the knockout fragment *waaC*U-FRT-*kan*-FRT-*waaF*D. Genes *waaC* and *waaF* in the chromosomes of HW001 or HW002 were removed using Red recombination. Plasmid pKD46 was first transformed into HW001 or HW002, and then the knockout fragment *waaC*U-FRT-*kan*-FRT-*waaF*D was amplified and transformed into the cells. The *waaC-waaF* locus was deleted from the chromosome through the recombination catalyzed by Red enzymes expressed by pKD46. The correct transformants were selected by growing cells on LB plates containing kanamycin, and plasmid pKD46 was cured by growing the cells at 42°C. The mutagenesis frequencies were around 12%. Next, plasmid pCP20 was transformed into the cells, and FLP recombinase was expressed to remove the *kan* gene inserted in the chromosome. Then, plasmid pCP20 was also cured by growing cells at 42°C, resulting in strains HWB01 and HWB02 [27,28]. There were no selection markers left on the chromosomes, and thus they can grow in medium without the addition of antibiotics.

### Isolation and analysis of crude Kdo_2_-lipid A, Kdo_2_-MPLA and Kdo_2_-P-MPLA from different *E*. *coli* strains

Lipids were isolated from 400 mL cultures of *E*. *coli* WBB06, HWB01, HWB02 cells at an OD_600_ of 1.5 using the Bligh-Dyer method [29]. Cells were harvested, washed once with phosphate-buffered saline (PBS) and stirred in a 76 mL-single-phase mixture of chloroform, methanol and water (1:2:0.8, v/v/v) for 1 h at room temperature. Lipids including Kdo_2_-lipid A or its derivatives were extracted from the cells. The supernatant was collected and converted into a two-phase Bligh-Dyer mixture of chloroform, methanol and water (2:2:1.8, v/v/v) by adding 20 mL chloroform and 20 mL water. Crude Kdo_2_-lipid A, Kdo_2_-MPLA or Kdo_2_-P-MPLA was isolated from the lower phase, dried with a rotary evaporator and stored at −20°C.

For TLC analysis, crude Kdo_2_-lipid A, Kdo_2_-MPLA or Kdo_2_-P-MPLA were dissolved in a mixture of chloroform and methanol (2:1, v/v), applied on silica gel 60 TLC plates, and separated in a glass chamber containing a solvent of chloroform, methanol, acetic acid and water (25:15:4:4, v/v/v/v). Then, the plates were dried, sprayed with 10% sulfuric acid in ethanol, and charred at 175°C to visualize the lipid bands [19,30,31]. The Rf values for the lipid bands on each TLC were determined.

For ESI/MS analysis, crude Kdo_2_-lipid A, Kdo_2_-MPLA or Kdo_2_-P-MPLA samples were dissolved in a mixture of chloroform and methanol (2:1, v/v) and analyzed using a Waters SYNAPT Q-TOF mass spectrometer equipped with an ESI source (Waters Corp., Milford, MA, USA) in the negative-ion mode [19,23]. Sodium formate solution was used for calibration. ESI/MS was carried out at -100 V, and the collisional activation of ions was performed at -6 V. Data acquisition and analysis were performed using MassLynx V4.1 software.

### Membrane permeability, hydrophobicity and auto-aggregation analyses of strains W3110, HW001, HW002, WBB06, HWB01 and HWB02

The membrane permeability, hydrophobicity and auto-aggregation of *E*. *coli* strains W3110, HW001, HW002, WBB06, HWB01 and HWB02 were determined according to published methods [30–32].

To evaluate membrane permeability, overnight cultures were harvested, washed twice with PBS, and resuspended in PBS to an OD_600_ of 0.5. The, 1.92 mL of the cell suspension was mixed with 80 μL of 1 mM N-phenylnaphthylamine (NPN, Sigma-Aldrich), and the fluorescence was immediately measured by a spectrofluorometer (Hitachi, Tokyo, Japan). The excitation wavelength, emission wavelength and slits used were 420 nm, 350 nm, and 5 nm, respectively. The permeability was indicated by the fluorescence absorption per OD_600_ value of the sample [33].

To measure the surface hydrophobicity, overnight cultures were harvested, washed twice with PBS (pH 7.4), and resuspended in PBS to an OD_600_ of approximately 1.0, which was recorded as A_0_. Then, 2 mL of the suspension was mixed with 800 μL of xylene and incubated for 3 h at 4°C. Then, the OD_600_ of the aqueous phase was measured and recorded as A. The value of [(A_0_-A)/A_0_]×100 represents the hydrophobicity.

For evaluate the auto-aggregation abilities of the tested strains, overnight cultures were harvested, resuspended in fresh liquid LB and adjusted to an OD_600_ of approximately 2.0, which was recorded as A_0_. Then, 12 mL of this suspension was added to a test tube and incubated at 22°C. After 24 h, the OD_600_ of the supernatant was monitored and recorded as A_i_. The value of [(A_0_-A_i_)/A_0_]×100 represents the auto-aggregation ability.

### Measurement of minimum inhibitory concentration of *E*. *coli* strains to antibiotics

Minimum inhibitory concentration (MIC) is defined as the concentration of antibiotics that significantly reduced the metabolic activity and inhibited visible cell growth (OD_600_≤0.15). MIC of novobiocin, erythromycin, and clarithromycin for strains W3110, HW001, HW002, WBB06, HWB01 and HWB02 was determined in sterile 96-well plates using microdilution method [32,34,35]. The mixture was composed of 100 μL bacterial suspension diluted in LB (OD_600_ around 0.02) and 100 μL antibiotics with different concentrations (0.98, 1.95, 3.91, 7.81, 15.6, 31.2, 62.5, 125, 250, 500, 1000 μg/mL), using LB as blank control. After incubation at 37°C for 24 h, the MIC and colony forming unit (CFU) values of bacterial cultures before and after treatment with antibiotics were determined. The experiments were performed twice in triplicate.

### Cell stimulation by whole cells of *E*. *coli* W3110, HW001, HW002, WBB06, HWB01 and HWB02

Whole-cell bacterial samples of *E*. *coli* W3110, HW001, HW002, WBB06, HWB01 and HWB02 were prepared according to published methods [36,37]. Briefly, bacterial cells were harvested at an OD_600_ of 1.5 and washed twice with PBS. Cell pellets were gently resuspended in PBS to approximately 1×10^8^ CFU/mL and subsequently diluted and incubated with 180 μL HKE-Blue hTLR4 cells (1.4×10^5^ cells/mL) in 96-well plates (Corning Costar) for 12 h. PBS containing no bacterial cells was used as blank control. HEK-Blue hTLR4 cells (InvivoGen) are engineered HEK293 cells in which the human TLR4 and NF-kB-inducible secreted embryonic alkaline phosphatase (SEAP) reporter genes were expressed. The cells were maintained in DMEM medium (4 mM L-glutamine, 4500 mg/L glucose, 1 mM sodium pyruvate, and 1500 mg/L sodium bicarbonate) containing 10% (v/v) heat-inactivated fetal bovine serum (HyClone), 4.5 g/L glucose, 2 mM L-glutamine, 50 U/mL-50μg/mL Pen-Strep (Gibco), 100 μg/mL normocin (InvivoGen) and 1×HEK-Blue selection (InvivoGen) in a humidified atmosphere of 5% CO_2_ at 37°C. The induction of hTLR4 signaling in HEK-Blue hTLR4 cells was assessed by measuring SEAP activity at OD_630_ using HEK-Blue^™^ Detection (InvivoGen), according to the manufacturer’s protocols. The experiments were performed twice in triplicate.

### Purification of different structures of Kdo_2_-MPLA and LPS for cell stimulation

Kdo_2_-lipid A, Kdo_2_-MPLA and Kdo_2_-pentaacyl-MPLA were extracted from 1 L overnight cultures of WBB06, HWB01 and HWB02, respectively, and purified using DEAE-cellulose columns (Whatman DE52) [15,30]. Briefly, crude lipid samples were dissolved in a mixture of chloroform, methanol and water (2:3:1, v/v/v) and loaded onto the DEAE-cellulose column equilibrated with the same solvent. The column was washed with increasing concentrations of ammonium acetate, and Kdo_2_-lipid A, Kdo_2_-MPLA or Kdo_2_-pentaacyl-MPLA samples were eluted with 360 mM ammonium acetate. The fractions containing Kdo_2_-lipid A, Kdo_2_-MPLA or Kdo_2_-pentaacyl-MPLA were pooled and converted into a two-phase mixture of chloroform, methanol and water (2:2:1.8, v/v/v). The lower phases containing the purified Kdo2-lipid A, Kdo_2_-MPLA or Kdo_2_-pentaacyl-MPLA were collected and dried.

LPS, PS-MPLA and PS-pentaacyl-MPLA were extracted from 1 L overnight cultures of W3110, HW001 and HW002 using a modified phenol/water method, and purified with RNase A, DNase I, and proteinase K treatment [15,19]. LPS samples were then subjected to Folch and Vogel extractions to further remove residual impurities [38,39].

About 32.8, 34.9, 29.7, 18.6, 20.3 and 19.5 mg purified LPS samples were obtained from WBB06, HWB01, HWB02, W3110, HW001 and HW002 cells, respectively. The purified samples were dissolved in PBS to the concentration of 1 mg/mL. To confirm the purity, DNA, RNA and proteins in the samples were measured by using NanoDrop 2000 Spectrophotometer (Thermo Scientific) [40,41], and 1 mg/mL of standard LPS (Sigma-Aldrich, Prod. No. L2630) was used as control.

### Stimulation of mouse RAW264.7 cells and human THP-1 cells using different LPS structures

The mouse macrophage cell line RAW264.7 and the human monocyte cell line THP-1 were obtained from ATCC. LPS stimulates these cells in similar manners [42,43]. RAW264.7 cells and THP-1 cells were grown in a humid atmosphere containing 5% CO_2_ at 37°C in DMEM medium and RPMI 1640 medium (2 mM L-glutamine, 10 mM HEPES, 1 mM sodium pyruvate, 4500 mg/L glucose, and 1500 mg/L sodium bicarbonate), respectively. Both media were supplemented with 10% (v/v) heat-inactivated fetal bovine serum (HyClone), 50 U/mL-50μg /mL Pen-Strep.

For this assay, 200 μL of RAW264.7 cells (1×10^5^ cells/mL) were prepared by incubating them in 96-well plates for 24 h, then the medium was replaced with fresh medium containing sonically dispersed purified LPS ligands diluted from the 1mg/mL stock suspensions to concentrations of 0.1, 1, 10, 100 ng/mL. The THP-1 cells (200 μL, 1×10^5^ cells/ml) were prepared by adding phorbol 12-myristate 13-acetate (PMA, Sigma-Aldrich) to differentiate the cells so that they could adhere to 96-well plastic plates [44]. After 48 h incubation, the medium was replaced with fresh medium containing sonically dispersed LPS ligands. After 24 h incubation in medium containing LPS ligands, the supernatants of both cell cultures were collected and stored at -80°C. Growth medium without any LPS ligands was used as blank control.

The levels of TNF-α, IL-6, IL-8 and RANTES released by the RAW264.7 cells or THP-1 cells were determined using enzyme-linked immunosorbent assay (ELISA) (R&D Systems) [45]. The experiments were performed twice in triplicate.

### Statistical analysis

All statistical analyses were performed using GraphPad Prism 5.0 software. Student’s t-test was used to analyze the difference between wild-type W3110 and *E*. *coli* mutants, and *p <0.05 and **p<0.01 were be considered as significant and statistically significant.

## Results

### Construction of *E*. *coli* mutants HWB01 and HWB02 that produce Kdo_2_-MPLA and Kdo_2_-pentaacyl-MPLA, respectively

Mutant strains HWB01 and HWB02 were constructed by deletion and integration of certain key genes related to LPS biosynthesis in the chromosome of *E*. *coli* W3110. HWB01 was constructed by integrating the *F*. *novicida lpxE* gene and deleting *lacI*, *waaC*, and *waaF* in the chromosome of wild type *E*. *coli* W3110 [27,28]; HWB02 was constructed by further deletion of *lpxM* in the chromosome of HWB01 [22]. The gene *lpxE* is found in *F*. *novicida* but not in *E*. *coli* [26], and the phosphatase LpxE that is encoded by *lpxE* can efficiently remove the 1-phosphate of lipid A in *E*. *coli*. Genes *waaC* and *waaF* encode heptosyltransferase WaaC and WaaF, respectively, and sequentially add two L-D-heptoses to the inner Kdo residue of Kdo_2_-lipid A in *E*. *coli* [26]. The acyltransferase LpxM, encoded by *lpxM*, adds a secondary acyl chain at the 3’-position of Kdo_2_-lipid A. Because *lpxE* was integrated in the *lacZ* site of the chromosome [18,19,26], *lacI* was also removed to release the control to the lactose operon. The correct deletion and replacement of genes in HWB01 and HWB02 were confirmed by using DNA sequencing.

**Fig 2:**
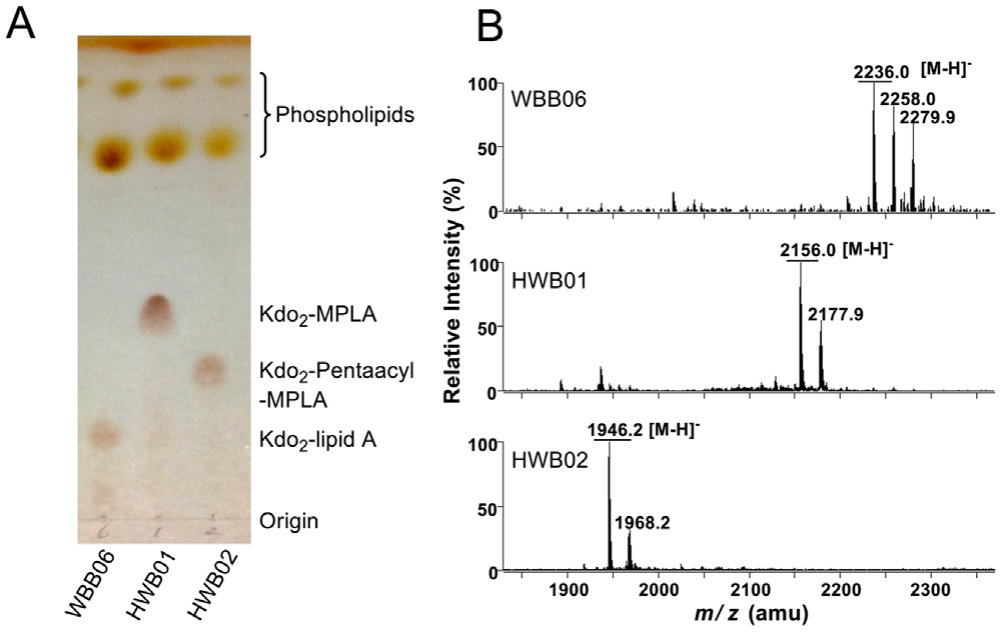
Crude lipids were isolated from WBB06, HWB01 and HWB02 cells and analyzed by TLC and ESI/MS. (A) TLC analysis. The lipid samples were dissolved in a solvent of chloroform and methanol (2:1, v/v) and developed in a solvent mixture of chloroform, methanol, acetic acid and water (25:15:4:4; v/v/v/v). (B) ESI/MS analysis.

To analyze the structure of LPS synthesized by the *E*. *coli* mutants HWB01 and HWB02, LPS was isolated from HWB01 and HWB02 and analyzed by TLC and ESI/MS (Fig 2) using WBB06 as a control. WBB06 can only synthesize Kdo_2_-lipid A because *waaC* and *waaF* have been inactivated in its chromosome (17). To determine the structural difference of LPS synthesized in HWB01 and HWB02, the total lipids were extracted from WBB06, HWB01 and HWB02 cells and separated by TLC [30] (Fig 2A). Two bands appeared at the top of the TLC plate for all samples. These bands represent phospholipids and migrated at similar rates, suggesting that the composition of the phospholipids in these three strains are similar. At the lower part of the TLC, however, there were bands that ran at different speeds for each sample. These bands may have been derived from Kdo_2_-lipid A, which usually migrates slower than phospholipids on TLC. Their different running speeds on TLC suggest that different structures of Kdo_2_-lipid A are produced by the WBB06, HWB01 and HWB02 cells. Rf values of Kdo_2_-lipid A molecules from HWB01, HWB02 and WBB06 were 0.416, 0.301 and 0.173, respectively, suggesting that Kdo_2_-lipid A molecules from HWB01, HWB02 are more hydrophobic due to the loss of the phosphate at the 1-position of lipid A. Kdo_2_-lipid A from HWB01 ran relatively faster than that from HWB02, suggesting the latter is more hydrophilic due to the loss of the second acyl chain at the 3’-position of lipid A.

To confirm their structures, Kdo_2_-lipid A samples extracted from *E*. *coli* WBB06, HWB01 and HWB02 were further analyzed by ESI/MS (Fig 2B). WBB06 Kdo_2_-lipid A created a major ionat m/z 2236.0 in the spectrum, which should be the molecular ion [M-H]^-^ of Kdo_2_-lipid A. Ions at m/z 2258.0 and 2279.9 were generated by the sodium adducts of Kdo_2_-lipid A. In the spectrum of the HWB01 sample, there was a major ion at *m/z* 2156.0, which is consistent with the [M-H]^-^ for Kdo_2_-MPLA; the ion at *m/z* 2177.9 might have been created by the sodium adduct of Kdo_2_-MPLA. In the spectrum of the HWB02 sample, there was a major ion at *m/z* 1946.2, which is consistent with the [M-H]^-^ for Kdo_2_-pentaacyl-MPLA, and the ion at *m/z* 1968.2 was created by the sodium adduct of Kdo_2_-pentaacyl-MPLA. Both ESI/MS and TLC analyses indicate that HWB01 synthesizes Kdo_2_-MPLA and HWB02 synthesizes Kdo_2_-pentaacyl-MPLA.

### HWB01 and HWB02 cells have higher membrane permeability, higher surface hydrophobicity and higher auto-aggregation ability than wild type *E*. *coli* cells

**Fig 3:**
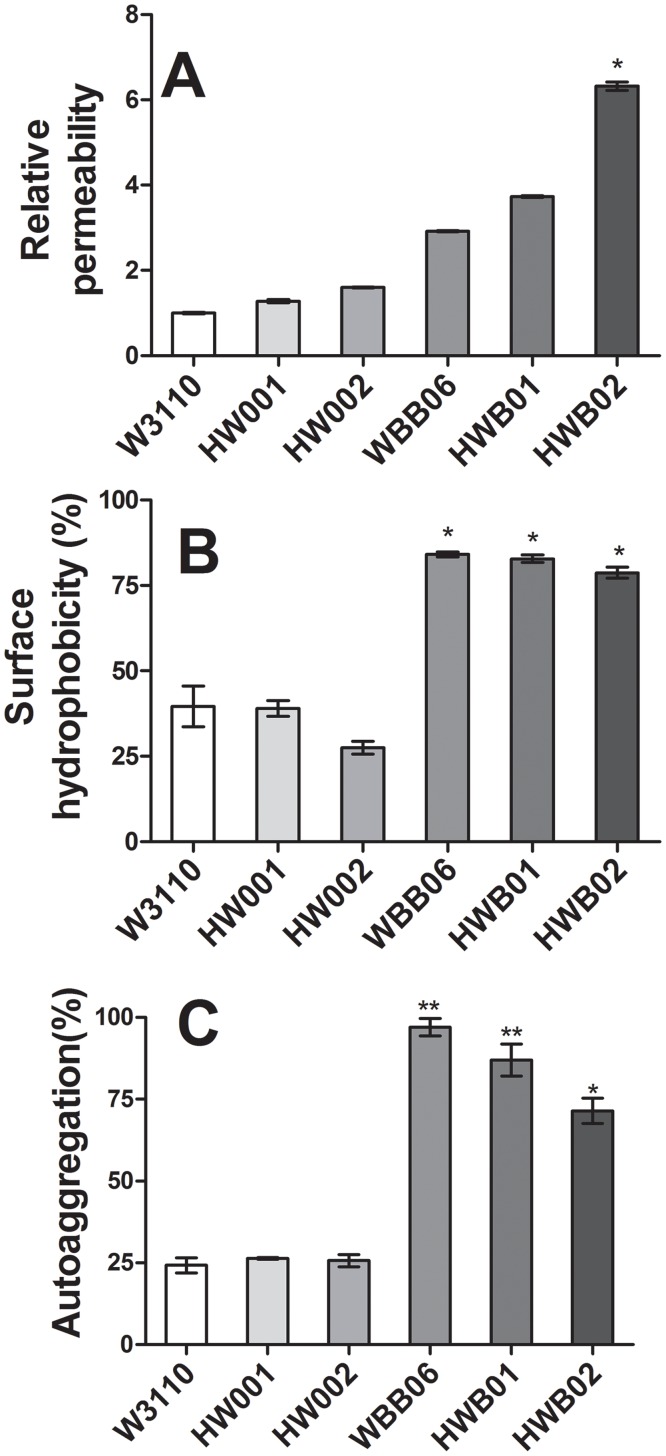
Comparison of the membrane permeability (A), surface hydrophobicity (B) and auto-aggregation (C) of *E*. *coli* strains W3110, HW001, HW002, WBB06, HWB01 and HWB02. Error bars indicate the standard deviations from three parallel samples. *, p <0.05; **, p<0.01.

LPS forms the major component of the outer membrane inmost Gram-negative bacteria, covering approximately 75% of the cell surface area. Therefore, LPS helps stabilize the outer membrane and protects it from chemical attack [46]. The outer membrane permeability, cell surface hydrophobicity, and auto-aggregation of HWB01 and HWB02 cells were evaluated, using W3110, HW001, HW002 and WBB06 as controls (Fig 3). Compared with W3110, membranes of HW001 and HW002 were only slightly more permeable, but the membrane permeability of HWB01 and HWB02 cells increased by 3- and 5-fold, respectively (Fig 3A). The sensitivity of W3110, WBB06, HW001, HW002, HWB01 and HWB02 cells to the antibiotics novobiocin, erythromycin, and clarithromycin was tested, and MIC values were listed in Table 2. At MIC, OD_600_ of the above cell cultures only reached up to 0.15; while OD_600_ of the controls without antibiotic treatment reached up to 1.0. At MIC, CFU values of the above strains only increased approximately 10 times; while CFU values of the controls without antibiotic treatment increased approximately 10 times (Table 3). Based on MIC and CFU values, the bacterial cell sensitivity to these antibiotics increased according to the order of W3110, HW001, HW002, WBB06, HWB01 and HWB02. The results indicate that LPS structure is an important determinant of antibiotic susceptibility. This is consistent with the results of membrane permeability, suggesting that the increased membrane permeability of HWB01 and HWB02 cells might lead to their increased sensitivity to antibiotics.

**Table 2:** Comparison of antibiotic sensitivities of *E*. *coli* strains W3110, WBB06, HW001, HWB01, HW002 and HWB02.

Antibiotic		MIC (μg/mL)				
	W3110	HW001	HW002	WBB06	HWB01	HWB02
Novobiocin	>500	250	62.5	31.25	3.91	1.95
Erythromycin	500	62.5	31.2	15.6	7.81	3.91
Clarithromycin	125	62.5	31.2	7.81	3.91	1.95

**Table 3:** CFU values (CFU/mL) of *E*. *coli* strains W3110, WBB06, HW001, HWB01, HW002 and HWB02 at OD_600_ of 0.02 before and after treatment with antibiotics of MIC as listed in Table 2.

	W3110	HW001	HW002	WBB06	HWB01	HWB02
Starting cultures	7.5×10^6^	5.2×10^6^	6.8×10^6^	4.5×10^6^	4.2×10^6^	4.0×10^6^
Novobiocin	6.7×10^7^	5.0×10^7^	7.6×10^7^	7.5×10^7^	2.1×10^7^	1.3×10^8^
Erythromycin	1.3×10^8^	1.0×10^8^	8.3×10^7^	7.9×10^7^	6.8×10^7^	1.2×10^8^
Clarithromycin	2.0×10^8^	1.0×10^8^	9.3×10^7^	1.6×10^8^	8.4×10^7^	9.0×10^7^
No antibiotics	2.2×10^9^	1.3×10^9^	9.6×10^8^	1.4×10^9^	9.4×10^8^	9.5×10^8^

The cell surface hydrophobicity of HW001 was almost the same as W3110 cells, but that of HW002 was slightly lower. However, the cell surface hydrophobicity of WBB06, HWB01 and HWB02 cells was significantly increased (Fig 3B). Loss of the hydrophilic polysaccharide of LPS in HWB01 and HWB02 cells might make these major molecules in the outer membrane more hydrophobic and thus increase the surface hydrophobicity of cells. The increased cell surface hydrophobicity might subsequently enhance the auto-aggregation of cells [47]. This is confirmed by the increased auto-aggregation of WBB06, HWB01 and HWB02 cells compared to that of W3110, HW001 and HW002 cells (Fig 3C). This increased aggregation may benefit the large-scale production of Kdo_2_-MPLA or Kdo_2_-pentaacyl-MPLA because aggregated cells are easier to collect.

### HWB01 and HWB02 cells showed less stimulating activities to HEK-Blue hTLR4 than wild type *E*. *coli* W3110

**Fig 4:**
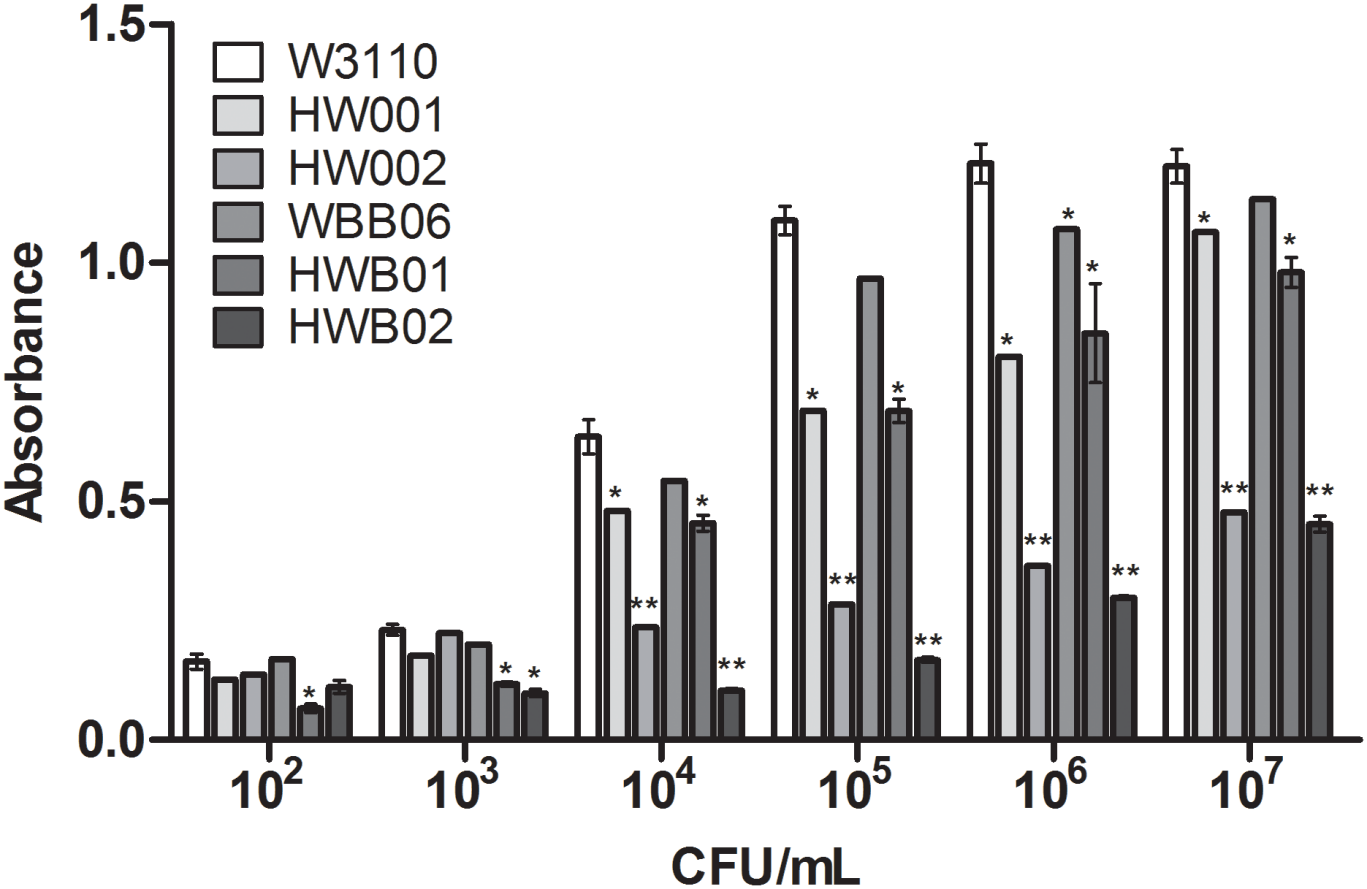
Comparison of TLR4 stimulation of whole cells of *E*. *coli* strains W3110, HW001, HW002, WBB06, HWB01 and HWB02. The SEAP activity was assessed by reading the OD_630_ with a microplate reader. *, p <0.05; **, p<0.01.

HEK-Blue hTLR4 cells expressing TLR4, MD-2, and CD14 were challenged with a range of CFU of W3110, HW001, HW002, WBB06, HWB01 or HWB02 cells, and the stimulating activities of these bacterial cells were determined by measuring the levels of the activator protein-1-dependent reporter SEAP in the mixtures. The stimulating activities of all of the strains were similar when less than 10^3^ CFU/mL bacteria were used, but quite different stimulatory activities were observed when more than 10^3^ CFU/mL bacteria were used (Fig 4). When more than 10^3^ CFU/mL bacteria were used, the stimulating activities of HWB01 and HWB02 cells were less than that of W3110 and WBB06. Overall, the stimulating activities of W3110 and WBB06 were similar, those of HW001 and HWB01 were similar, and those of HW002 and HWB02 were also similar. For example, in a mixture containing 10^6^ CFU/mL bacteria, the A_630_ reached 1.21 for W3110 cells, 1.07 for WBB06, 0.80 for HW001 cells, 0.85 for HWB01, 0.36 for HW002 and 0.30 for HWB02 cells. These results indicate that the stimulating activity of *E*. *coli* cells to HEK-Blue hTLR4 cells was dramatically affected by the lipid A portion of the LPS. The results further show that the absence of polysaccharide on the outer membrane did not impact the binding efficiency of LPS with the TLR4 complex. The reduction in the TLR4 activation of HWB01 and HWB02 suggests that Kdo_2_-MPLA or Kdo_2_-pentaacyl-MPLA might be used for developing novel vaccine adjuvants [36,48].

### Comparison of stimulation activities of LPS, PS-MPLA, PS-pentaacyl-MPLA, Kdo_2_-lipid A, Kdo_2_-MPLA and Kdo_2_-pentaacyl-MPLA

**Fig 5:**
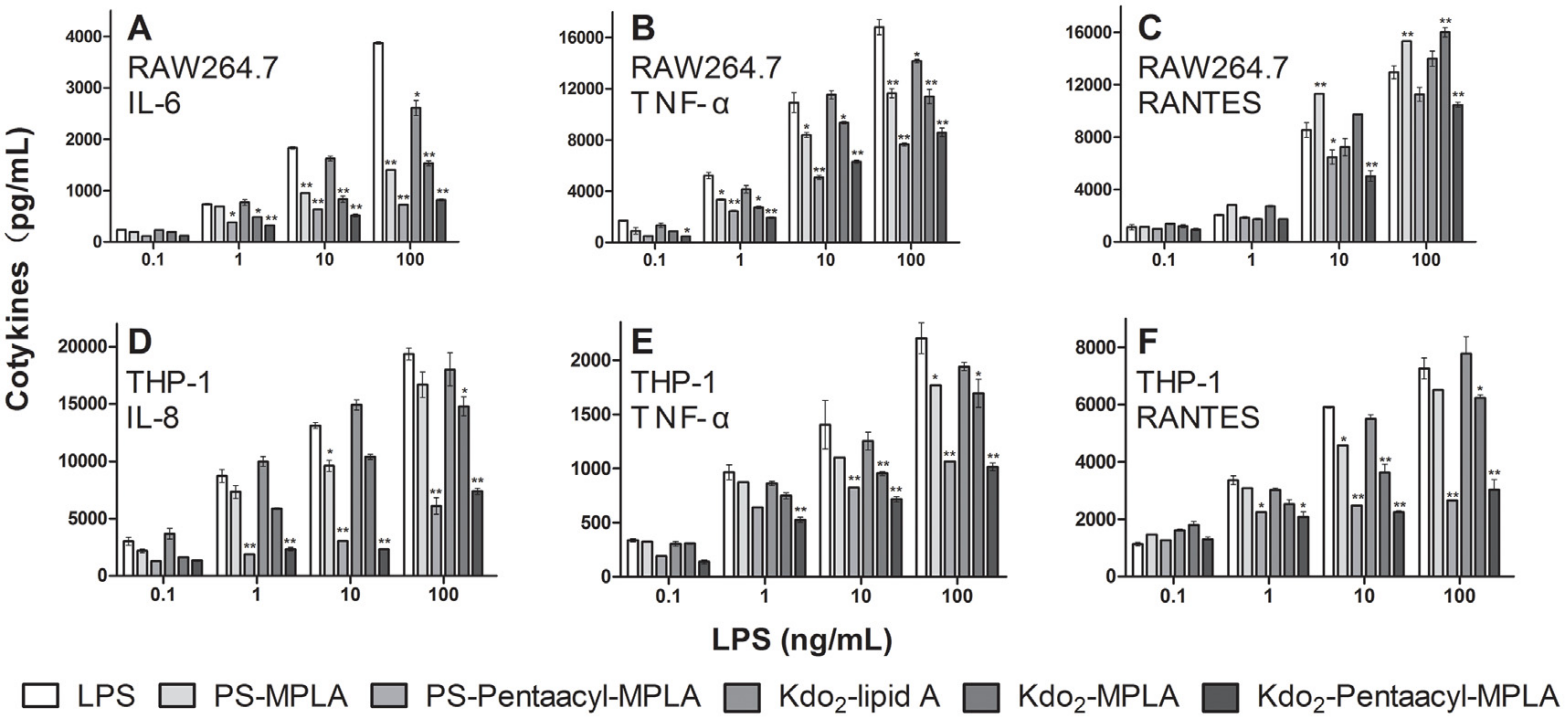
Immunogenicity of purified LPS, PS-MPLA, PS-pentaacyl-MPLA, Kdo_2_-lipid A, Kdo_2_-MPLA and Kdo_2_-pentaacyl-MPLA from W3110, HW001, HW002, WBB06, HWB01 and HWB02, respectively. Levels of the MyD88 pathway cytokines IL-6 (A), IL-8 (D), TNF-α (B, E), and the TRIF pathway cytokine RANTES (C, F) were measured by ELISA. Error bars indicate the standard deviations from three parallel samples. *, p <0.05; **, p<0.01.

To further investigate the effect of structural changes on the stimulatory activity of LPS, purified LPS, PS-MPLA, PS-pentaacyl-MPLA, Kdo_2_-lipid A, Kdo_2_-MPLA and Kdo_2_-pentaacyl-MPLA were used to stimulate mouse macrophage cell line RAW264.7 or human monocyte cell line THP-1, using fresh growth medium as blank control (Fig 5). Levels of the cytokines TNF-α, IL-6, IL-8 or RANTES in the reaction mixtures were determined by ELISA. The cytokine levels were different because of the differences in the structure and concentration of LPS included in the mixtures, but they all increased as the concentrations of LPS ligands increased (Fig 5).

In RAW264.7 cell mixtures, LPS and Kdo_2_-lipid A induced the highest levels of TNF-α and IL-6 and the second highest levels of RANTES; PS-MPLA and Kdo_2_-MPLA induced the second highest levels of TNF-α and IL-6, and the highest levels of RANTES; Kdo_2_-MPLA and Kdo_2_-Pentaacyl-MPLA induced the least levels of TNF-α, IL-6 and RANTES (Fig 5A–5C). In THP-1 cell mixtures, LPS and Kdo_2_-lipid A induced the highest levels of TNF-α, IL-6 and RANTES; PS-MPLA and Kdo_2_-MPLA induced the second highest levels of TNF-α, IL-6 and RANTES; Kdo_2_-MPLA and Kdo_2_-Pentaacyl-MPLA induced the least levels of TNF-α, IL-6 and RANTES (Fig 5D–5F).

Under all tested circumstances, the stimulating activity of Kdo_2_-lipid A was similar to that of LPS, the stimulating activity of Kdo_2_-MPLA was similar to that of PS-MPLA, and the stimulating activity of Kdo_2_-pentaacyl-MPLA was similar to that of PS-pentaacyl-MPLA. Kdo_2_-pentaacyl-MPLA induced the least stimulation in all mixtures. The stimulating activity of Kdo_2_-MPLA or Kdo_2_-pentaacyl-MPLA was always lower than LPS, suggesting that Kdo_2_-MPLA or Kdo_2_-pentaacyl-MPLA may be good candidates for vaccine adjuvant development [49].

## Discussion

In this work, *E*. *coli* mutants HWB01 and HWB02 were constructed by deleting and integrating key genes related to LPS biosynthesis so that they were able to synthesize Kdo_2_-MPLA and Kdo_2_-pentaacyl-MPLA, respectively. With the loss of hydrophilic polysaccharide, the 1-phosphate group or the 3’-secondery acyl chain, the structural changes to LPS not only influenced the membrane permeability and cell surface hydrophobicity of HWB01 and HWB02 cells but also decreased the ability of LPS to activate the TLR4/MD-2 receptor of mammalian cells. The stimulating ability of Kdo_2_-pentaacyl-MPLA was lower than that Kdo_2_-MPLA, suggesting the importance of the secondary acyl chain at 3’-position of lipid A. The secondary acyl chain at the 3’-position is bound deeply into the MD-2 component of the TLR4/MD-2 complex [50], therefore, secondary deacylation at the 3’-position of Kdo_2_-MPLA might alter the dimerization of TLR4/MD-2 and consequently inhibit its activation. Kdo_2_-MPLA and Kdo_2_-pentaacyl-MPLA could moderately stimulate RAW264.7 and THP-1 cells and had biological activities comparable to MPLA [51]. Therefore, Kdo_2_-MPLA and Kdo_2_-pentaacyl-MPLA may be good candidates for immune-pharmacological exploitations, vaccine adjuvant engineering, and anti-inflammatory intervention investigation (38).

In cells, MPLA is usually connected to polysaccharides. To prepare MPLA, LPS has to be first isolated and then hydrolyzed. The isolation and quantification of LPS are difficult because of the large size and micro-heterogeneity of the molecule, and hydrolyzing LPS can damage the structure of MPLA; thus, the efficiency and quality of MPLA prepared from LPS are limited. Kdo_2_-MPLA and Kdo_2_-pentaacyl-MPLA, however, can be directly isolated from HWB01 and HWB02 cells without hydrolysis, and their small size and micro-homogeneity make them easier to purify. HWB01 and HWB02 were constructed by marker-less deletion and integration into the chromosome to facilitate the production of Kdo_2_-MPLA and Kdo_2_-pentaacyl-MPLA by fermentation. Future studies should focus on optimizing the growth conditions of HWB01 and HWB02 or genetically modifying the strains for large-scale industrial fermentation. The findings in this study may have important implications for the development of future vaccine adjuvants.

## References

[1] Raetz CR, Whitfield C (2002) Lipopolysaccharide endotoxins. Annu Rev Biochem 71: 635–700. 12045108 10.1146/annurev.biochem.71.110601.135414 PMC2569852

[2] Wang X, Quinn PJ (2010) Endotoxins: lipopolysaccharides of gram-negative bacteria. Subcell Biochem 53: 3–25. 10.1007/978-90-481-9078-2_1 20593260

[3] Nikaido H (2003) Molecular basis of bacterial outer membrane permeability revisited. Microbiol Mol Biol Rev 67: 593–656. 14665678 10.1128/MMBR.67.4.593-656.2003 PMC309051

[4] Baker PJ, Hraba T, Taylor CE, Myers KR, Takayama K, Qureshi N, et al (1992) Structural features that influence the ability of lipid A and its analogs to abolish expression of suppressor T cell activity. Infect Immun 60: 2694–2701. 1535339 10.1128/iai.60.7.2694-2701.1992 PMC257223

[5] Miller SI, Ernst RK, Bader MW (2005) LPS, TLR4 and infectious disease diversity. Nat Rev Microbiol 3: 36–46. 15608698 10.1038/nrmicro1068

[6] Beutler B, Rietschel ET (2003) Innate immune sensing and its roots: the story of endotoxin. Nat Rev Immunol 3: 169–176. 12563300 10.1038/nri1004

[7] Bohannon JK, Hernandez A, Enkhbaatar P, Adams WL, Sherwood ER (2013) The immunobiology of toll-like receptor 4 agonists: from endotoxin tolerance to immunoadjuvants. Shock 40: 451–462. 10.1097/SHK.0000000000000042 23989337 PMC3919163

[8] Thompson BS, Chilton PM, Ward JR, Evans JT, Mitchell TC (2005) The low-toxicity versions of LPS, MPL adjuvant and RC529, are efficient adjuvants for CD4+ T cells. J Leukoc Biol 78: 1273–1280. 16204643 10.1189/jlb.0305172

[9] Mata-Haro V, Cekic C, Martin M, Chilton PM, Casella CR, Mitchell TC. (2007) The vaccine adjuvant monophosphoryl lipid A as a TRIF-biased agonist of TLR4. Science 316: 1628–1632. 17569868 10.1126/science.1138963

[10] Gaekwad J, Zhang Y, Zhang W, Reeves J, Wolfert MA, Boons GJ. (2010) Differential induction of innate immune responses by synthetic lipid a derivatives. J Biol Chem 285: 29375–29386. 10.1074/jbc.M110.115204 20634284 PMC2937970

[11] Coler RN, Bertholet S, Moutaftsi M, Guderian JA, Windish HP, Baldwin SL, et al (2011) Development and characterization of synthetic glucopyranosyl lipid adjuvant system as a vaccine adjuvant. PLoS One 6: e16333 10.1371/journal.pone.0016333 21298114 PMC3027669

[12] Park BS, Song DH, Kim HM, Choi BS, Lee H, Lee JO. (2009) The structural basis of lipopolysaccharide recognition by the TLR4-MD-2 complex. Nature 458: 1191–1195. 10.1038/nature07830 19252480

[13] Galanos C, Luderitz O, Westphal O (1969) A new method for the extraction of R lipopolysaccharides. Eur J Biochem 9: 245–249. 5804498 10.1111/j.1432-1033.1969.tb00601.x

[14] Garcon N (2010) Preclinical development of AS04. Methods Mol Biol 626: 15–27. 10.1007/978-1-60761-585-9_2 20099118

[15] Wang X, Zhang C, Shi F, Hu X (2010) Purification and characterization of lipopolysaccharides. Subcell Biochem 53: 27–51. 10.1007/978-90-481-9078-2_2 20593261

[16] Whitfield C, Trent MS (2014) Biosynthesis and export of bacterial lipopolysaccharides. Annu Rev Biochem 83: 99–128. 10.1146/annurev-biochem-060713-035600 24580642

[17] Reynolds CM, Raetz CR (2009) Replacement of lipopolysaccharide with free lipid A molecules in *Escherichia coli* mutants lacking all core sugars. Biochemistry 48: 9627–9640. 10.1021/bi901391g 19754149 PMC2782650

[18] Chen J, Tao G, Wang X (2011) Construction of an *Escherichia coli* mutant producing monophosphoryl lipid A. Biotechnol Lett 33: 1013–1019. 10.1007/s10529-011-0521-z 21246253

[19] Han Y, Li Y, Chen J, Tan Y, Guan F, Wang X. (2013) Construction of monophosphoryl lipid A producing *Escherichia coli* mutants and comparison of immuno-stimulatory activities of their lipopolysaccharides. Mar Drugs 11: 363–376. 10.3390/md11020363 23434832 PMC3640385

[20] Wang X, Karbarz MJ, McGrath SC, Cotter RJ, Raetz CR (2004) MsbA transporter-dependent lipid A 1-dephosphorylation on the periplasmic surface of the inner membrane: topography of *Francisella novicida* LpxE expressed in Escherichia coli. J Biol Chem 279: 49470–49478. 15339914 10.1074/jbc.M409078200 PMC2552400

[21] Raetz CR, Guan Z, Ingram BO, Six DA, Song F, Wang X, et al (2009) Discovery of new biosynthetic pathways: the lipid A story. J Lipid Res 50 Suppl: S103–108. 10.1194/jlr.R800060-JLR200 18974037 PMC2674688

[22] Wang X, Quinn PJ, Yan A (2015) Kdo_2_-lipid A: structural diversity and impact on immunopharmacology. Biol Rev Camb Philos Soc 90: 408–427. 10.1111/brv.12114 24838025 PMC4402001

[23] Raetz CR, Garrett TA, Reynolds CM, Shaw WA, Moore JD, Smith DC, et al (2006) Kdo_2_-Lipid A of *Escherichia coli*, a defined endotoxin that activates macrophages via TLR-4. J Lipid Res 47: 1097–1111. 16479018 10.1194/jlr.M600027-JLR200

[24] Murphy RC, Raetz CR, Reynolds CM, Barkley RM (2005) Mass spectrometry advances in lipidomica: collision-induced decomposition of Kdo_2_-lipid A. Prostaglandins Other Lipid Mediat 77: 131–140. 16099398 10.1016/j.prostaglandins.2004.09.004 PMC4535423

[25] Wang X, Quinn PJ (2010) Lipopolysaccharide: Biosynthetic pathway and structure modification. Prog Lipid Res 49: 97–107. 10.1016/j.plipres.2009.06.002 19815028

[26] Brabetz W, Muller-Loennies S, Holst O, Brade H (1997) Deletion of the heptosyltransferase genes *rfaC* and *rfaF* in *Escherichia coli* K-12 results in an Re-type lipopolysaccharide with a high degree of 2-aminoethanol phosphate substitution. Eur J Biochem 247: 716–724. 9266718 10.1111/j.1432-1033.1997.00716.x

[27] Datsenko KA, Wanner BL (2000) One-step inactivation of chromosomal genes in *Escherichia coli* K-12 using PCR products. Proc Natl Acad Sci U S A 97: 6640–6645. 10829079 10.1073/pnas.120163297 PMC18686

[28] Cherepanov PP, Wackernagel W (1995) Gene disruption in *Escherichia coli*: TcR and KmR cassettes with the option of Flp-catalyzed excision of the antibiotic-resistance determinant. Gene 158: 9–14. 7789817 10.1016/0378-1119(95)00193-a

[29] Bligh EG, Dyer WJ (1959) A rapid method of total lipid extraction and purification. Can J Biochem Physiol 37: 911–917. 13671378 10.1139/o59-099

[30] Wang L, Hu X, Tao G, Wang X (2012) Outer membrane defect and stronger biofilm formation caused by inactivation of a gene encoding for heptosyltransferase I in *Cronobacter sakazakii* ATCC BAA-894. J Appl Microbiol 112: 985–997. 10.1111/j.1365-2672.2012.05263.x 22353600

[31] Wang J, Ma W, Wang Z, Li Y, Wang X (2014) Construction and characterization of an *Escherichia coli* mutant producing Kdo_2_-lipid A. Mar Drugs 12: 1495–1511. 10.3390/md12031495 24633251 PMC3967223

[32] Wang Z, Wang J, Ren G, Li Y, Wang X (2015) Influence of Core Oligosaccharide of Lipopolysaccharide to Outer Membrane Behavior of Escherichia coli. Mar Drugs 13: 3325–3339. 10.3390/md13063325 26023839 PMC4483631

[33] Helander IM, Mattila-Sandholm T (2000) Fluorometric assessment of gram-negative bacterial permeabilization. J Appl Microbiol 88: 213–219. 10735988 10.1046/j.1365-2672.2000.00971.x

[34] Andrews JM (2001) Determination of minimum inhibitory concentrations. J Antimicrob Chemother 48 Suppl 1: 5–16. 11420333 10.1093/jac/48.suppl_1.5

[35] Nweze EI, Ghannoum A, Chandra J, Ghannoum MA, Mukherjee PK (2012) Development of a 96-well catheter-based microdilution method to test antifungal susceptibility of *Candida biofilms* . J Antimicrob Chemother 67: 149–153. 10.1093/jac/dkr429 21990050 PMC3236055

[36] Needham BD, Carroll SM, Giles DK, Georgiou G, Whiteley M, Trent MS (2013) Modulating the innate immune response by combinatorial engineering of endotoxin. Proc Natl Acad Sci U S A 110: 1464–1469. 10.1073/pnas.1218080110 23297218 PMC3557076

[37] Hankins JV, Madsen JA, Giles DK, Childers BM, Klose KE, Brodbelt JS, et al (2011) Elucidation of a novel *Vibrio cholerae* lipid A secondary hydroxy-acyltransferase and its role in innate immune recognition. Mol Microbiol 81: 1313–1329. 10.1111/j.1365-2958.2011.07765.x 21752109 PMC3178793

[38] Folch J, Lees M, Sloane Stanley GH (1957) A simple method for the isolation and purification of total lipides from animal tissues. J Biol Chem 226: 497–509. 13428781

[39] Hirschfeld M, Ma Y, Weis JH, Vogel SN, Weis JJ (2000) Cutting edge: repurification of lipopolysaccharide eliminates signaling through both human and murine toll-like receptor 2. J Immunol 165: 618–622. 10878331 10.4049/jimmunol.165.2.618

[40] Desjardins P, Conklin D (2010) NanoDrop microvolume quantitation of nucleic acids. J Vis Exp. 10.3791/2565 PMC3346308 21189466

[41] Desjardins P, Hansen JB, Allen M (2009) Microvolume spectrophotometric and fluorometric determination of protein concentration. Curr Protoc Protein Sci Chapter 3: Unit 3 10. 10.1002/0471140864.ps0310s55 19235138

[42] Weinstein SL, June CH, DeFranco AL (1993) Lipopolysaccharide-induced protein tyrosine phosphorylation in human macrophages is mediated by CD14. J Immunol 151: 3829–3838. 7690817

[43] Hambleton J, Weinstein SL, Lem L, DeFranco AL (1996) Activation of c-Jun N-terminal kinase in bacterial lipopolysaccharide-stimulated macrophages. Proc Natl Acad Sci U S A 93: 2774–2778. 8610116 10.1073/pnas.93.7.2774 PMC39708

[44] Janusch H, Brecker L, Lindner B, Alexander C, Gronow S, Heine H, et al (2002) Structural and biological characterization of highly purified hepta-acyl lipid A present in the lipopolysaccharide of the *Salmonella enterica sv*. *Minnesota* Re deep rough mutant strain R595. J Endotoxin Res 8: 343–356. 12537693 10.1179/096805102125000678

[45] Li Y, Wang Z, Chen J, Ernst RK, Wang X (2013) Influence of lipid A acylation pattern on membrane permeability and innate immune stimulation. Mar Drugs 11: 3197–3208. 10.3390/md11093197 24065161 PMC3806461

[46] Delcour AH (2009) Outer membrane permeability and antibiotic resistance. Biochim Biophys Acta 1794: 808–816. 10.1016/j.bbapap.2008.11.005 19100346 PMC2696358

[47] Rahman M, Kim WS, Kumura H, Shimazaki K (2008) In vitro effects of bovine lactoferrin on autoaggregation ability and surface hydrophobicity of bifidobacteria. Anaerobe 14: 73–77. 10.1016/j.anaerobe.2008.01.002 18313948

[48] Cullen TW, O'Brien JP, Hendrixson DR, Giles DK, Hobb RI, Thompson SA, et al (2013) EptC of Campylobacter jejuni mediates phenotypes involved in host interactions and virulence. Infect Immun 81: 430–440. 10.1128/IAI.01046-12 23184526 PMC3553815

[49] Oblak A, Jerala R (2015) The molecular mechanism of species-specific recognition of lipopolysaccharides by the MD-2/TLR4 receptor complex. Mol Immunol 63: 134–142. 10.1016/j.molimm.2014.06.034 25037631

[50] Ohto U, Fukase K, Miyake K, Shimizu T (2012) Structural basis of species-specific endotoxin sensing by innate immune receptor TLR4/MD-2. Proc Natl Acad Sci U S A 109: 7421–7426. 10.1073/pnas.1201193109 22532668 PMC3358893

[51] Sasaki H, White SH (2008) Aggregation behavior of an ultra-pure lipopolysaccharide that stimulates TLR-4 receptors. Biophys J 95: 986–993. 10.1529/biophysj.108.129197 18375521 PMC2440428

[52] Datsenko KA, Wanner BL (2000) One-step inactivation of chromosomal genes in *Escherichia coli* K-12 using PCR products. Proc Natl Acad Sci U S A 97: 6640–6645. 10829079 10.1073/pnas.120163297 PMC18686

